# Private Asylum for the Insane

**Published:** 1860-03

**Authors:** 


PRIVATE ASYLUM FOR THE INSANE.—We are glad to have it in our power to announce the opening of a private asylum for the insane, which combines the advantages of a rural and healthy location and easy access, with suitable construction and internal arrangements for the reception and treatment of this class of the afflicted. The conception and execution of the plan thus carried out, are due to the energy of the gentleman, Dr. Robert A. Given, who is both proprietor and medical superintendent of this new establishment.

Of his fitness for the responsible post which Dr. Given has now assumed, we can speak with some confidence, based on a knowledge of his having been the medical assistant of Dr. Kirkbride in the Pennsylvania Hospital for the Insane, during a period of three years, and of his having, as physician to the Eastern Penitentiary for a longer time, exerted a most salutary influence as well in the treatment of cases of insanity occurring in that institution, as in the still more important matter of prevention of the disease by wisely devised measures of hygiene. Dr. Given brings with him to his present undertaking not only experience, but also abundant zeal, firmness, and integrity of purpose, and a deep conviction of the responsibility which he incurs in the discharge of the difficult and delicate duties which are henceforth to devolve on him.

Clifton Hall is the name by which the new establishment is designated. The grounds which it overlooks, and on which it stands, are twenty-five acres in extent, of which five are woodland: they are abundantly supplied with excellent water. Clifton Hall is situated in Delaware County, Pennsylvania, about seven miles west of Philadelphia, and is easily reached both by turnpike and railroad, thus uniting the indispensable requisites of location in the country with facility for its inmates occasionally visiting a large city. "It is designed," as we learn from a circular now on our desk, "to accommodate about fifty patients, twenty-five of each sex, and no efforts have been spared to render it both cheerful and healthy. To the eye, the external appearance is decidedly pleasing, while the arrangements for heating, ventilation, and the admission of light, will compare favorably with those of larger establishments. The opportunities for classification are believed to be ample, and the cubic space allotted to each inmate has been declared by competent observers to be even more than either health or convenience demands."

We learn, also, that provision has been made for that form of insanity called *mania a potu*, which has been denied admission in some other establishments. "For this form of disease special arrangements exist, and, after recovery, those who may wish to remain for a time, in the hope of overcoming their unfortunate propensity, are at liberty to do so, provided they VOLUNTARILY submit to the established rules of the institution."

It is argued in favor of a private asylum, like the present one, that fifty patients living as a family, and being more frequently under the eye of the superintendent, will be more likely to show, unveiled, "the exact nature and starting-point of their morbid mental manifestations, be better protected against the not unfrequent indiscretions of attendants, and have every rational desire more fully gratified than would be possible in hospitals containing five or six times that number." Dr. Given has successfully obviated the objections which used to be brought against private lunatic asylums in England, viz., the want of a responsible supervisory body, in his having obtained a Board of Supervision composed, at present, of Drs. Bell, Carson, and Dickson, the Rev. Wm. R. Breed, and John Jenkins, D.D., Judge Strong, Hon'ble Wm. A. Porter, Herman Cope, Francis Tete, Morton McMichael, Chas. Kelly, and Oborn Levis, Esquires. Further strong collateral support in favor of the soundness of the working details in Clifton Hall is furnished in the fact, that Dr. Given has secured the aid of Dr. Charles Evans, so well and favorably known as Physician to the Frankford Asylum for the Insane, as consulting physician. "Besides, whenever it is so desired by his friends, the patient may still retain the services of his ordinary medical adviser."

